# Effect of green tea extract (*Camellia sinensi*s) on the spermatic parameters of Wistar rats submitted or not to testicular heat shock

**DOI:** 10.1590/1984-3143-AR2019-0049

**Published:** 2020-05-28

**Authors:** Joane Isis Travassos Vieira, Taylane Alves da Silva, Williane Maria Pereira Barbosa, Gabriela Lima de Azevêdo, Lúcia Cristina Pereira Arruda, Maria Madalena Pessoa Guerra, Pierre Castro Soares, Ellen Cordeiro Bento da Silva

**Affiliations:** 1 Departamento de Medicina Veterinária, Universidade Federal Rural de Pernambuco, Recife, PE, Brasil; 2 Departamento de Morfologia e Fisiologia Animal, Universidade Federal Rural de Pernambuco, Recife, PE, Brasil

**Keywords:** catechins, heat, spermatozoa

## Abstract

The aim of this study was to evaluate the effect of green tea extract (GTE) on the spermatic parameters of Wistar rats, submitted or not to testicular heat shock (HS). For this, 48 animals were treated according to the experimental groups (G1: not exposed to HS and untreated; G2: exposed to HS and untreated; G3: not exposed to HS and treated with GTE; G4: exposed to HS and treated with GTE). Subgroups of rats were euthanized on days 15, 30, and 60 to recover the spermatozoa. The total motility (TM), vigor, spermatic morphology and concentration, mitochondrial membrane potential, plasma membrane integrity, and acrosome integrity (ACi) were analyzed. The TM was higher in G1 and G3 than in G2 and G4 on day 30, and higher in G4 on day 60. The overall means of TM and vigor were higher in G1 and G3 than in G2 and G4, as well as TM on day 60. For the morphology, G2 and G4 were lower than G1 and G3 on day 15, and G4 was lower than G1 and G3 on day 30. Moreover, in G1 and G3 morphology was higher on days 15 and 30, and in G4 it was lower on day 30, with the overall means being higher in G1 and G3 than in G2 and G4, as well as on days 15 and 60 compared to day 30. The overall mean of ACi, on day 30, was lower than on days 15 and 60 for all the groups. Therefore, HS is shown to be widely deleterious to the gametes, and the daily administration of 100 mg/kg green tea extract does not improve the spermatic parameters of Wistar rats, submitted or not to testicular HS, although it leads to better recovery of spermatic motility and morphology at 60 days.

## Introduction

Thermal stress affects humans and all other animal species (Nóbrega et al., 2011; Santos et al., 2015), since it can generate alterations in physiological and behavioral parameters, including the reproductive potential of males (Gabaldi and Wolf, 2002; Cruz et al., 2011). Testicular temperatures below or above the appropriate temperature are harmful to gonadal functions, meaning thermoregulation mechanisms are crucial to avoid these situations (Durairajanayagam et al., 2015).

Exposure of the testicles to heat may trigger significant alterations in semen quality (Abshenas et al., 2011; Hamilton et al., 2016) and a reduction in gonadal weight and volume. Recovery is dependent on the specific conditions to which the animal was subjected (Setchell, 1998). This is because germ cells, present in male gonads, are sensitive to thermal variations and may even deteriorate when exposed to body temperature (Absalan et al., 2008).

Similarly, Sertoli cells suffer from the action of heat and, because of their responsibility for nourishing and developing germ cells, seminal quality and sperm viability are further impaired by the increase in temperature (Garcia, 2006), culminating in spermatogenesis impairment (Moreira et al., 2001). The epididymis is also a target of the deleterious effects of heat shock (Moreira et al., 2001), evidenced by sperm damage (Banks et al., 2005; Hamilton et al., 2016).

Some important causes of testicular hyperthermia are varicoceles, cryptorchidism, body posture or position, clothing, obesity, lifestyle, occupation, fever episodes, and ambient seasonal temperature changes (Durairajanayagam et al., 2015). During thermal stress, even for a short time, reactive oxygen species (ROS) are produced in greater quantity, resulting in oxidative damage to germ cells (Ishii et al., 2005). To control excessive ROS production and restore the state of balance, a powerful antioxidant system is required (Vasconcelos et al., 2007). In this context, natural antioxidants have been highlighted for maintaining the integrity of the sperm and fertility (Zanchi et al., 2015).

Phenolic compounds stand out for their high antioxidant power, which is due to their chemical structure, rich in hydroxyls and aromatic rings (Cerqueira et al., 2007). Among these compounds, flavonoids are antioxidants from natural sources found in leaves, fruits, and seeds (Angelo and Jorge, 2007), with high potential to sequester free radicals, chelate metals, and inhibit lipoperoxidation (Schmitz et al., 2005; Barreiros et al., 2006).

The *Camellia sinensis* plant, from which green tea is produced, is rich in flavonoid polyphenols and catechins (Schmitz et al., 2005). Epigallocatechin gallate (EGCG) is the main polyphenol in green tea (Tachibana et al., 2004), demonstrating rapid absorption and distribution throughout the body, as well as a long half-life, which is reduced when the substance is isolated (Schmitz et al., 2005).

Based on the above, the current study aimed to evaluate the effects of green tea extract on the spermatic parameters of Wistar rats submitted or not to testicular heat shock.

## Material and methods

The study was carried out in the Physiology, Pathology, and Andrology laboratories of the Federal Rural University of Pernambuco (UFRPE - Brazil). The experimental protocol was approved by the Ethics Committee for Animal Experimentation of UFRPE, under process number CEUA/UFRPE 059/2015.

A total of 48 adult Wistar rats (*Rattus norvegicus*, *var. Albinus*), from the bioterium of the Department of Animal Morphology and Physiology (DMFA/UFRPE), were kept in an environment with a temperature of 23±1 °C, 50% humidity, and light-dark cycle of 12 h. Water and rodent feed were offered *ad libitum* during the experimental period. At 120 days of age, the animals were divided by random probabilistic sampling into four experimental groups, each with 12 subjects (G1: not exposed to heat shock and untreated; G2: exposed to heat shock and untreated; G3: not exposed to heat shock and treated with 100 mg/kg of green tea extract; G4: exposed to heat shock and treated with 100 mg/kg of green tea extract).

The green tea extract (China-Hong Kong/Pharma Nostra® São Paulo, Brazil) was weighed and diluted in distilled water (200 mg/mL), so that each animal in the G3 and G4 groups received 100 mg/kg of the aqueous solution daily, based on 50 mg/kg of epigallocatechin gallate present in the extract used (Ding et al., 2015). On day 0, the animals of the G2 and G4 groups were anesthetized intraperitoneally with 80 mg/kg of 10% Ketamine Hydrochloride (Syntec/São Paulo, Brazil) and 6mg/kg of 2% Xylazine hydrochloride (Syntec/São Paulo, Brazil), and placed in a support for immersion of their testicles in water at 43 °C for 15 min (Queiroz et al., 2013). On the same day, after recovery from anesthesia, the administration of aqueous solution of green tea extract or distilled water was initiated, according to the experimental group, once a day, by oral gavage.

The treatment continued for 60 days, during which the animals were weighed daily on a digital scale (Tomato®/China) for correction of the administered dose of the extract. Subgroups of animals (n=04 per group) were euthanized, according to Queiroz et al. (2013), with intraperitoneal administration of an overdose of the anesthetic Thiopental Sodium 0.5 g, 50 mg/kg (Tiopentax® Cristália/São Paulo, Brazil), 15, 30, and 60 days after the beginning of the experiment. All the right epididymides were removed and rigorously washed in Tris buffer solution [3.605 g Tris Hydroxyamyl aminomethane (Invitrogen), 2.024 g citric acid (Sigma Aldrich^®^), 1.488 g fructose (Sigma Aldrich®), and distilled water to 100 mL solution; pH 6.8]. In the same solution (1 mL), spermatozoa were recovered by the flotation technique (Lima, 2013), with the aid of tweezers and a scalpel.

The recovered gametes were evaluated for total motility (TM) and vigor, morphology and concentration, mitochondrial membrane potential (MMP), plasma membrane integrity (PMi), and acrosome integrity (ACi). The TM and sperm vigor were determined through subjective evaluation under an optical microscope (Leica DM500; 100X), depositing an aliquot (10 μL) of the recovered sperm sample on a previously heated slide under a coverslip (37 °C). Sperm motility was rated from 0 to 100% and vigor from 0 to 5 (CBRA, 2013).

The evaluation of spermatic morphology was performed using the moist chamber technique (CBRA, 2013). For this purpose, aliquots of semen were diluted in saline formalin solution at a ratio of 1:100 and 200 cells analyzed under a phase contrast microscope (Olympus), with an increase of 1000X. The same semen samples diluted in formalin were used to determine the sperm concentration by the Neubauer chamber method, under a phase contrast microscope (Olympus), with an increase of 400X (CBRA, 2013).

For analysis of plasma membrane integrity (PMi), dual-staining was used with carboxyfluorescein diacetate (CFD, Sigma Aldrich^®^) and propidium iodide (PI;Sigma Aldrich^®^), according to Silva et al. (2012). On a slide covered with a coverslip, a total of 200 cells were analyzed under an epifluorescence microscope (Carl Zeiss, Germany, 400X), using a DBP 580-630 nm emission filter and DBP 485/20 nm excitation filter. The gametes were classified as presenting an intact plasma membrane, when stained in green, or injured, when stained in red.

The mitochondrial membrane potential (MMP) was determined using the fluorochrome JC-1 (Molecular probes by Life Technologies), according to Silva et al. (2012). Under a slide and cover slip, 200 spermatozoa were analyzed in an epifluorescence microscope (Carl Zeiss, Germany, 400X), using a BP 450-490 nm excitation filter and LP 515 nm emission filter. The cells were classified as presenting high or low MMP when the intermediate piece fluoresced in orange or green, respectively.

Acrosome integrity (ACi) was assessed using the fluorescein isothiocyanate probe conjugated with *peanut agglutinin* (FITC-PNA; Sigma Aldrich®) (Silva et al., 2012). From each slide, 200 spermatozoa were analyzed, with a 515-nm LP emission filter and 450-490-nm BP excitation filter, under an epifluorescence microscope (Carl Zeiss; Germany, 1000X). These were classified as presenting intact acrosome when the acrosomal region fluoresced in green, and reacted acrosome when it did not fluoresce.

The data were analyzed according to ProcMixed for mixed models (SAS, 1999), with collection days as repeated measurements over time (15, 30, and 60 days). First, the data were analyzed in relation to the presence of normality of the residues (Shapiro-Wilk). Logarithmic or square root transformations (x+1) were necessary when the normality premise was not met. All variables with percentage responses were submitted to transformation by arcosene of X (%). The statistical model includes fixed treatment effect (T), days (D), and interactions (TxD). The comparison of means was performed by the Student-Newman-Keuls test at the 5% probability level.

## Results

It was observed that the percentage of mobile spermatozoa (Table 1) was higher (P<0.05) in the G1 and G3 groups than in the G2 and G4, at 30 days. In addition, on day 60, the G4 presented superior motility (P<0.05) compared to days 15 and 30. Similarly, the general means were higher (P<0.05) in the G1 and G3 groups than in the G2 and G4, as well as being higher on day 60 (P<0.05) compared to 15 and 30 days.

**Table 1:** Mean and standard deviations of spermatic motility (%) of Wistar rats submitted or not to testicular heat shock and treated or not with 100 mg/kg of green tea extract at different times.

	**Days**			
**GROUP**	**D15**	**D30**	**D60**	**Overall mean**
G1	63.75±17.02aA	86.25±4.79aA	80.00±20.41aA	76.67A
G2	50.00±40.93aA	28.33±18.93aB	67.50±20.21aA	42.17B
G3	61.25±20.16aA	82.50±8.66aA	60.00±7.07aA	67.92A
G4	20.00±13.54bA	8.67±7.09bB	67.50±18.48aA	31.33B
Overall mean	45.69b	49.13b	68.75a	

Different lowercase letters on the lines and different capital letters in the columns show significant differences (P<0.05) between the times and groups, respectively. G1: not stressed and untreated; G2: stressed and untreated; G3: not stressed and treated; G4: stressed and treated.

The spermatic vigor (Table 2) demonstrated higher overall means (P<0.05) in the G1 and G3 groups compared to the G2 and G4. The percentage of morphologically normal cells (Table 3) was higher (P<0.05) in the G1 and G3 groups than in the G2 and G4 on day 15, whereas on day 30, the percentage was lower in the G4 (P<0.05) than in the G1 and G3. In addition, this parameter was higher (P<0.05) in the G1 and G3 on days 15 and 30 than on day 60, and in G4 it was lower (P<0.05) on day 30 than on days 15 and 60. In the same way, the G1 and G3 groups presented higher overall means (P<0.05) than the G2 and G4, with the latter being lower (P<0.05) than the G2. Moreover, at 30 days the overall mean was lower (P<0.05) than at 15 and 60 days.

**Table 2:** Mean and standard deviations of the spermatic vigor (0-5) of Wistar rats submitted or not to testicular heat shock and treated or not with 100 mg/kg of green tea extract at different times.

	**Days**			
**GROUP**	**D15**	**D30**	**D60**	**Overall mean**
G1	3.00±0.00Aa	3.00±0.91Aa	2.75±0.50Aa	2.92A
G2	2.13±1.44Aa	1.50±1.00Aa	2.75±0.50Aa	2.13B
G3	3.00±0.82Aa	3.13±0.48Aa	3.00±0.82Aa	3.04A
G4	2.50±0.58Aa	1.25±0.96Aa	2.63±0.48Aa	2.13B
Overall mean	2.66a	2.22a	2.78a	

Different capital letters in the columns show significant differences (P<0.05) between the groups. G1: not stressed and untreated; G2: stressed and untreated; G3: not stressed and treated; G4: stressed and treated.

**Table 3:** Mean and standard deviations of morphologically normal spermatozoids (%) of Wistar rats submitted or not to testicular heat shock and treated or not with 100 mg/kg of green tea extract at different times.

	**Days**			
**GROUP**	**D15**	**D30**	**D60**	**Overall mean**
G1	91.50±4.88aA	85.99±8.05aA	67.78±3.54bA	81.76A
G2	52.53±26.67aB	35.42±27.70aAB	67.29±2.68aA	51.75B
G3	88.88±3.86aA	88.88±5.94aA	72.95±8.18bA	83.57A
G4	51.88±13.55aB	8.22±7.09bB	55.91±16.88aA	38.67C
Overall mean	71.19a	54.63b	65.98a	

Different lowercase letters on the lines and different capital letters in the columns show significant differences (P<0.05) between the times and groups, respectively. G1: not stressed and untreated; G2: stressed and untreated; G3: not stressed and treated; G4: stressed and treated.

For the spermatic concentration (Table 4), it was found that the overall means were higher (P<0.05) in the G1 and G3 groups than in the G2 and G4, and that on day 15 the number of cells was higher than on days 30 and 60, independent of the experimental group. Regarding the acrosome integrity (Table 5), the overall mean on day 30 was lower (P<0.05) than on days 15 and 60. No statistical differences (P>0.05) were observed for plasma membrane integrity (Table 6) or mitochondrial membrane potential (Table 7).

**Table 4:** Mean and standard deviations of the spermatic concentration (10^6^ spz/mL) of Wistar rats submitted or not to testicular heat shock and treated or not with 100 mg/kg of green tea extract at different times.

		**Days**		
**GROUP**	**D15**	**D30**	**D60**	**Overall mean**
G1	26.31±1.91Aa	11.19±7.14Aa	6.81±3.70Aa	14.77A
G2	13.88±5.78Aa	1.58±0.29Aa	2.88±0.66Aa	6.88B
G3	17.31±7.26Aa	15.13±10.22Aa	4.88±3.23Aa	12.44A
G4	13.75±5.42Aa	2.94±2.41Aa	3.19±1.81Aa	6.63B
Overall mean	17.81a	8.28b	4.44b	

Different lowercase letters on the lines and different capital letters in the columns show significant differences (P<0.05) between the times and groups, respectively. G1: not stressed and untreated; G2: stressed and untreated; G3: not stressed and treated; G4: stressed and treated.

**Table 5:** Mean and standard deviation of spermatozoid acrosome integrity (%) of Wistar rats submitted or not to testicular heat shock and treated or not with 100 mg/kg of green tea extract at different times.

	**Days**			
**GROUP**	**D15**	**D30**	**D60**	**Overall mean**
G1	21.25±6.69Aa	10.25±4.84Aa	37.00±19.27Aa	22.83A
G2	12.88±5.06Aa	12.38±4.59Aa	18.25±7.50Aa	14.50A
G3	12.88±5.57Aa	16.38±10.43Aa	37.50±23.16Aa	22.25A
G4	21.00±18.40Aa	5.13±4.33Aa	14.63±10.49Aa	13.58A
Overall mean	17.00a	11.03b	26.84a	

Different lowercase letters on the lines show significant differences (P<0.05) between the times. G1: not stressed and untreated; G2: stressed and untreated; G3: not stressed and treated; G4: stressed and treated.

**Table 6:** Mean and standard deviations of plasma membrane integrity of spermatozoids (%) of Wistar rats submitted or not to testicular heat shock and treated or not with 100 mg/kg of green tea extract at different times.

	**Days**			
**GROUP**	**D15**	**D30**	**D60**	**Overall mean**
G1	3.67±2.75Aa	4.83±2.36Aa	8.49±4.38Aa	5.04A
G2	4.00±3.50Aa	5.83±6.66Aa	6.12±1.25Aa	4.50A
G3	15.45±14.35Aa	4.88±2.81Aa	4.75±2.18Aa	8.36A
G4	5.00±4.95Aa	2.25±0.87Aa	8.63±6.09Aa	5.29A
Overall mean	6.55a	3.84a	7.00a	

G1: not stressed and untreated; G2: stressed and untreated; G3: not stressed and treated; G4: stressed and treated.

**Table 7:** Mean and standard deviations of high mitochondrial spermatozoid membrane potential (%) of Wistar rats submitted or not to testicular heat shock and treated or not with 100 mg/kg of green tea extract at different times.

	**Days**			
**GROUP**	**D15**	**D30**	**D60**	**Overall mean**
G1	37.67±34.24Aa	53.38±20.17Aa	29.31±27.56Aa	34.54A
G2	32.00±26.00Aa	22.50±9.73Aa	18.83±18.41Aa	18.38A
G3	44.83±38.89Aa	57.63±22.17Aa	34.74±25.45Aa	39.31A
G4	38.63±26.26Aa	28.50±24.44Aa	29.00±26.92Aa	29.67A
Overall mean	31.13a	37.31a	22.98a	

G1: not stressed and untreated; G2: stressed and untreated; G3: not stressed and treated; G4: stressed and treated.

## Discussion

The current study evaluated the effects of green tea extract on the spermatic parameters of Wistar rats, submitted or not to testicular heat shock, at three different times (15, 30, and 60 days). Significant decreases were found in motility, vigor, morphology, and sperm concentration in semen samples recovered from epididymis of the animals exposed to testicular thermal stress, particularly marked after 30 days, independent of the treatment. The acrosome integrity overall mean on day 30 was smaller than at the other times. However, the use of green tea extract possibly led to better recovery of the spermatic parameters from 60 days after the heat shock.

According to the descriptions above, testicular heat shock was detrimental to the maintenance of gamete quality, regardless of administration of green tea extract, which is rich in antioxidants (Schmitz et al., 2005), with day 30 representing the moment of maximum impairment. The deleterious action of the high temperature on seminal parameters has been described in previous studies, and evidenced by a reduction in the volume, concentration, morphology, motility, vigor, and plasma membrane integrity of semen samples (Coelho et al., 2006; Ghasemi et al., 2009; Abshenas et al., 2011; Ding et al., 2015). This is a consequence of the sensitivity of testicular germ cells to thermal variations (Absalan et al., 2008). However, adequate maintenance of these parameters is imperative for fertility (Severo, 2009; Arruda et al., 2015).

It is worth noting that the decrease in sperm parameters is correlated not only with thermal modifications, but also with many other stress factors, as well as the consequently higher serum glucocorticoids levels (Kumar et al., 2019; Rizk et al., 2020). Activation of the hypothalamic-pituitary-adrenal axis by stress determines, directly or indirectly, disturbances in the hypothalamic-pituitary-testicular axis, with a reduction in testosterone levels, commitment of spermatogenesis, and sperm quality (Kumar et al., 2019). It was evidenced that chronic exposure of Wistar rats to predator-induced psychosocial stress caused a significant reduction in total sperm count, and motile and progressive sperm, which was more noticeable after 52 days (Uwejigho et al., 2019). Thus, the animal manipulation during the investigation, may have contributed to reducing sperm parameters for all the groups, over the time.

The spermatogenic cycle of Wistar rat is around 46 (Almeida et al., 2000) to 52 days (Lucinda et al., 2010). This fact indicates that the 30th day after testicular heat shock, especially in the G4, was the critical moment for some spermatic parameters, since the gametes were obtained from injured gonads and epididymis, without time to recover their potential. In contrast, at 60 days a new spermatogenic cycle had finished, justifying the improvement in seminal quality from this time.

Consolidating the previous idea, Babaei et al. (2007) found a decline in spermatogenesis up to day 30 after testicular heat shock (43 °C/15min) in mice, through histopathological analysis. Similarly, Abshenas et al. (2011) described that partial recovery of Wistar rat sperm parameters after testicular heating (42 °C/20 min), did not occur before 42 days except in those treated with 500 or 750 mg/kg of green tea extract. These animals presented reestablished sperm motility and concentration in a short time; improvements that were also observed in the rats treated and not exposed to thermal stress.

The pronounced recovery of spermatic motility and morphology, presented at 60 days in the stressed and supplemented group (G4), although slow, could be attributed to the high amount of catechins present in the green tea extract, which are effective in preventing lipid peroxidation (Zanchi et al., 2015). However, the beneficial effect of oral or intraperitoneal doses of green tea extract on spermatic physiology and fertility is dose-dependent (Chandra et al., 2011). Thus, this compound can act as a protective testicular factor at moderate concentrations (Abshenas et al., 2011), or as a castrative agent at high concentrations (Das and Karmakar, 2015).

The intraperitoneal administration of 50 mg/kg EGCG improves rat testicular function after ionizing radiation (Ding et al., 2015), as well as 100 mg/kg green tea extract after exposure to lead acetate (Miraj et al., 2015). Nevertheless, as previously reported, for testicular heat shock the beneficial effect of green tea was obtained with higher concentrations (500 and 750 mg/kg), resulting in improvement in seminal parameters in rodents (Abshenas et al., 2011). Based on this information and on the poor results obtained with the use of the extract, it is possible that the concentration of 100 mg/kg was insufficient to protect the testicular and epididymis functions against thermal stress.

Despite the absence of prominent positive results in the groups treated with green tea extract, compared with untreated groups, the antioxidant activity of EGCG, the main catechin of green tea, has already been validated as, in addition to sequestering ROS, it prevents damage to biomolecules such as lipids and proteins (Yang et al., 2009; Mereles and Hunstein, 2011). Therefore, the effects of green tea extract may be relevant to spermatozoa which have membranes rich in polyunsaturated fatty acids, making them more susceptible to lipid peroxidation in the presence of elevated ROS levels (Alvarez et al., 1987), whose production is intensified by testicular heat stress (Ikeda et al., 1999).

It is worth noting that EGCG is the most abundant and potent catechin of green tea, and that its half-life is greater when in association with the other constituents of this tea (Schmitz et al., 2005). Moreover, the cost of green tea extract is much lower than that of isolated EGCG (Senger et al., 2010). Thus, the promising role and economic feasibility of green tea extract administration are noticeable in the maintenance or recovery of male fertility. This is beginning to be demonstrated in testicular tissue stressed by heat, especially because of the protective effect against oxidative damage (Awoniyi et al., 2011).

Based on the information above, studies should be carried out to identify the ideal concentrations of green tea extract to be used for each specie and situation. This could help to resolve the pathogenic effects of testicular oxidative stress induced by heat injuries, irradiation, ischemia, and inflammation, among others (Abshenas et al., 2011; Al-Maghrebi et al., 2012; Ding et al., 2015).

## Conclusion

Heat stress is shown to be widely deleterious to the gametes, and the daily administration of 100 mg/kg green tea extract does not improve the spermatic parameters of Wistar rats, submitted or not to testicular heat stress, although it leads to better recovery of spermatic motility and morphology 60 days after the stress.
